# Editorial: Anti-cancer effects of natural products against reproductive cancers

**DOI:** 10.3389/fphar.2023.1132795

**Published:** 2023-01-16

**Authors:** Rosy Iara Maciel Azambuja Ribeiro, Bonglee Kim

**Affiliations:** ^1^ Experimental Pathology Laboratory, Midwest Campus, Federal University of São João del-Rei, Divinópolis, Brazil; ^2^ Department of Pathology, College of Korean Medicine, Kyung Hee University, Seoul, South Korea

**Keywords:** Pharmacognosy, Phytochemistiy, reproductive system, Antitumoral, traditional medicine

## Introduction

Reproductive cancer treatments vary, among other factors, in terms of when the diagnosis occurs and the type of cancer. In women, the most common reproductive cancers are cervical, ovarian, uterine, vaginal, vulvar, and breast (occasionally referred to as reproductive cancer). In turn, prostate, testicular and penile cancers represent cancers of the male reproductive system (IARC, 2022).

The signs, symptoms, risk factors, prevention strategies, and treatments for reproductive cancers vary. Chemotherapy, surgery, and radiotherapy are the most common treatments, but there are limitations, and metastases are challenging (Wayteck et al., 2014).

In traditional and modern societies, natural products have a long history of use as food and medicine. They are also sources of new bioactive compounds. It is known that natural products can correct the imbalance in the human body caused by numerous diseases, such as diabetes, neurodegenerative disorders, and cancer. They represent an outstanding source of structural scaffolds with a high degree of diversity that may provide promising chemical agents for treating or even curing cancer. Anticancer effects of natural products on cancer-related proteins, epigenetic factors, and reactive oxygen species have been demonstrated (Akhtar et al., 2020).

This research topic focused on natural products, traditional medicine, and herbal remedies for the treatment of reproductive cancers. Five experimental research articles on reproductive cancer are presented here. Of these, three are about female cancer: cervical, endometrial and ovarian and two are about male cancer, both about prostate cancer. There are also two reviews on prostate cancer.

## In this section, we briefly introduce each paper

Cervical cancer is the most common type of gynecologic cancer (IARC, 2022), ranking second in cancer incidence in developing countries (Motoki et al., 2015). Cervical cancer is less prevalent in high-income countries because cervical cancer screening is widely available and effective, and the human papillomavirus (HPV) vaccine is also widely available (Petersen et al., 2022). Jiang et al.suggested that Resveratrol may potentiate the effect of cisplatin cis-DDP (cis-dichlorodiamine-platinum (II) in the treatment of cervical cancer. They demonstrated that this effect depended on Sirtuin 3 (SIRT3) and was related to the activation of the enzyme MnSOD antioxidant and increased H_2_O_2_ content.

Ovarian cancer ranks second in death rates for gynecological cancers (American Cancer Society, 2022). The standard treatments are cytoreductive surgery and platinum-based chemotherapy for gynecological cancers. Less than 49% of diagnosed patients live 5 years. Natural products and small molecules had antitumor effects by regulating miRNA. Furthermore, Icariin, a flavonoid derived from Epimedium, showed influence on several physiological and pathological processes through the modulation of specific miRNAs. Fu et al. investigated the activity of Icariin against ovarian cancer *in vitro* (SKOV-3 cells) and *in vivo* using female BALB/C nude mice. Icariin inhibits the growth of ovarian cancer SKOV-3 cells *in vitro* and *in vivo*. Furthermore, Icariin induced cell cycle arrest and apoptosis in SKOV-3 cells by blocking TNKS2/Wnt/β-catenin signaling through the tumor suppressor miR-1-3p.

In turn, surgical treatment is the choice for endometrial carcinoma of the uterine body (UCEC). However, adjuvant therapies can manage low- or medium-risk UCEC. Natural compounds used in traditional Chinese medicine have therapeutic effects with potential tumor selectivity and low cytotoxicity. Lin et al. Found a new prognostic model to predict the patient’s survival cycle accurately and conveniently, besides a new treatment approach for UCEC. To investigate capsaicin’s potential mechanism and therapeutic target in the treatment of UCEC, they analyzed transcriptome sequencing data from the TCGA database and single-cell sequencing data from the GEO database. Among the many pharmacological effects of capsaicin is the regulation of genes involved in carcinogenesis, which affects survival, angiogenesis, and metastasis. This study provided a new treatment strategy for UCEC, new genetic markers for UCEC prognosis, and a rationale for targeting capsaicin as a drug treatment for UCEC. However, no relevant clinical trial results were found. Consequently, it was impossible to determine whether UCEC has a definitive therapeutic effect.

Prostate cancer (PCa) ranks second in incidence and fifth in mortality rate across the globe. In addition, PCa tends to increase more due to increased life expectancy, especially in developed countries (Gco, 2022). Screening for candidate compounds for the treatment of prostate cancer pointed to 5′-epiequisetin (Eeq), a tetramic acid derivative isolated from the fungus *Fusarium equiseti* derived from a marine sponge, especially for the androgen-independent human prostate cancer lineage, PC-3. Wang et al. demonstrated that Eeq suppressed PC-3 proliferation by inhibiting the PI3K/Akt signaling pathway; it also restricted cell migration related to the suppression of the beta-catenin/cadherin signaling pathway. It induced apoptosis through the DR5 signaling pathway and suppressed prostate cancer growth *in vivo*. The results of this study may help in the search for compounds with similar structures for the development of anti-PCa drugs.

The widely used clinical treatment for PCa is androgen deprivation therapy (ADT). The literature indicates that, due to a decrease in sexual function resulting from the treatment, patients can develop depression. This situation aggravates the patient’s case since psychological problems and stress can promote the development of PCa and metastasis. There is evidence that lipid metabolism is involved in psychological depression or chronic stress through glucocorticoid and inflammatory pathways. Based on this, Li et al. showed that CSS, a classic Shugan formula from Traditional Chinese Medicine, reprogrammed lipid metabolism in stressed BALB/c nude mice exposed to unpredictable chronic mild stress (CUMS) with CaP. They found significant variations of 62 lipids in the serum lipid profiles of these mice, with 32 lipids showing recovery after treatment with CSS.

Reviews intend to guide future research. In this topic, there are two reviews on prostate cancer. One study concerns the combination of anticancer drugs and natural products. The other review highlights the importance of nutraceuticals for disease control and management, including during chemotherapy. Although prostate cancer is common, the use of natural products in conjunction with anticancer drugs is uncommon, according to Cheon and Ko. They reviewed 19 *in vitro* and 10 *in vivo* studies on antitumor drugs and natural products for prostate cancer. Most studies used a single extract from mushrooms or herbs. PC-3, LNCaP, C4-2, DU-145, and 22Rv were the most used cancer cell lines. Co-administered drugs included docetaxel and doxorubicin. Co-administering anticancer drugs boost natural substances' effectiveness.

Chopra et al. highlight the need to improve drug treatment, increasing specificity and reducing systemic toxicity. An individual response that causes resistance to treatment and increasing drug doses that lead to accumulation and systemic toxicity causes immunodeficiency and long-term adverse effects and constitute challenges for treating the cancer patient. A diet rich in vegetables and fruits can, to some extent, prevent the development of PCa. Nanoparticles improve phytochemical delivery, stability, and availability. Nanotechnology offers powerful treatment techniques to target malignant cells. This review examines the efficacy of phytochemicals in liposomes, nanoparticles, and nano cells, including lycopene, curcumin, genistein, silybin, plumbagin, oleuropein, and shikonin. Encapsulating nutraceuticals in nanoparticles improves delivery, stability, and availability.

## Conclusion

Natural products were utilized to correct the imbalance in the human body caused by various diseases, including diabetes, neurodegenerative diseases, and cancer. They contain promising chemical agents that make cancers less destructive and potentially curable. In reproductive cancer, natural products have demonstrated anticancer effects on cancer-related proteins, epigenetic factors, and reactive oxygen species. Using various experimental models, it is worthwhile to investigate the anticancer effects and mechanisms of natural products intended for clinical use.
